# The Procoagulant Effect of COVID-19 on the Thrombotic Risk of Patients with Hip Fractures Due to Enhanced Clot Strength and Fibrinolysis Shutdown

**DOI:** 10.3390/jcm10153397

**Published:** 2021-07-30

**Authors:** Andreas G. Tsantes, Dimitrios V. Papadopoulos, Ioannis G. Trikoupis, Stavros Goumenos, Daniele Piovani, Konstantina A. Tsante, Andreas F. Mavrogenis, Aristeidis G. Vaiopoulos, Panagiotis Koulouvaris, Georgios K. Nikolopoulos, Panayiotis J. Papagelopoulos, Stefanos Bonovas, Argirios E. Tsantes

**Affiliations:** 1 Laboratory of Haematology and Blood Bank Unit, “Attiko” Hospital, School of Medicine, National and Kapodistrian University of Athens, 12462 Athens, Greece; ktsante@yahoo.com (K.A.T.); avaiopoulos@gmail.com (A.G.V.); atsantes@yahoo.com (A.E.T.); 2 Department of Orthopaedic Surgery, University of Pittsburgh, Pittsburgh, PA 15237, USA; di_papadopoulos@yahoo.gr; 3 First Department of Orthopaedics, National and Kapodistrian, School of Medicine, University of Athens, 12462 Athens, Greece; gtrikoupis@hotmail.com (I.G.T.); stgoumenos@gmail.com (S.G.); afm@otenet.gr (A.F.M.); info@drkoulouvaris.gr (P.K.); pjporthopedic@gmail.com (P.J.P.); 4 Department of Biomedical Sciences, Humanitas University, Pieve Emanuele, 20090 Milan, Italy; dpiovani@hotmail.com; 5 IRCCS Humanitas Research Hospital, Rozzano, 20089 Milan, Italy; 6 Department of Epidemiology and Public Health, Medical School, University of Cyprus, Nicosia 1678, Cyprus; gnikolopoulos@gmail.com

**Keywords:** COVID-19, hip fractures, coagulopathy, thrombotic risk, fibrinolysis shutdown

## Abstract

Introduction: Coronavirus disease 2019 (COVID-19) in patients with hip fractures is associated with increased incidence of venous thromboembolism (VTE). The purpose of this study was to evaluate the hemostatic alterations of COVID-19 that are associated with a higher thrombotic risk using rotational thromboelastometry (ROTEM). Methods: A retrospective observational study was performed including 20 COVID-19 patients with hip fractures. To compare the coagulopathy of patients with mild COVID-19 and hip fractures with the coagulopathy associated with each of these two conditions separately, we used two previously recruited groups of patients; 198 hip fracture patients without COVID-19 and 21 COVID-19 patients without hip fractures. The demographics, clinical parameters, conventional coagulation parameters and ROTEM findings of the three groups were analyzed and compared. Results: COVID-19 hip fracture patients had higher amplitude of clot firmness at 10 min (*p* < 0.001), higher alpha angle (*p* < 0.001), higher lysis index at 60 min (*p* < 0.001), and shorter clot formation time (*p* < 0.001) than non-COVID-19 hip fracture patients, indicating increased clot strength and impaired fibrinolysis due to COVID-19. The value of lysis index at 60 min (99%) in COVID-19 patients with hip fractures was consistent with fibrinolysis shut down. Multivariable linear regression analysis further confirmed that COVID-19 resulted in increased amplitude of clot firmness at 10 min (*p* < 0.001), increased maximum clot firmness (*p* < 0.001), increased lysis index at 60 min (*p* < 0.001) and increased alpha angle (*p* < 0.001), but significantly shortened clot formation time (*p* < 0.001). Discussion: The higher thrombotic risk in COVID-19 patients with hip fractures is characterized by increased clot strength and fibrinolysis shutdown, as shown by ROTEM findings. Further prospective studies are warranted to evaluate the need for modification of thromboprophylaxis to balance the hemostatic derangements of COVID-19 patients with hip fractures.

## 1. Introduction

The COVID-19 pandemic has transformed trauma care services, with a paucity of elective surgeries compensating for the limited operating capacity. Although there was a significant reduction in major orthopedic trauma due to reduced population activity, the incidence of fragility fractures such as hip fractures remained unchanged [1,2,3,4]. These injuries are common in elderly people, a population highly susceptible to COVID-19. Although the exact impact of COVID-19 on the morbidity and mortality of these patients is not clear, it appears that the additive effect of COVID-19 leads to poor outcomes and a low survival rate [5]. Both conditions have a high risk for certain adverse events such as venous thromboembolism, thus the overall risk for such complications is significantly increased.

Although with the advent of modern thromboprophylaxis the incidence of venous thromboembolism after hip fractures has been substantially decreased, the rate is still relatively high [6]. Several causes contribute to the thrombotic risk of hip fractures including endothelial damage and immobilization, while enhanced thrombin generation due to trauma-induced coagulopathy has also been hypothesized [7,8]. Numerous studies have also shown that COVID-19 results in a prothrombotic state with devastating clinical implications due to arterial and venous thromboembolic events [9,10,11,12]. There is a large body of emerging evidence regarding the pathophysiologic mechanisms involved in this prothrombotic state. Viral infection-associated inflammation and endothelial dysfunction have been theorized as the leading causes of prothrombotic state, while impaired fibrinolysis has been identified as an important mechanism for COVID-19 associated coagulopathy [9,10]. 

Laboratory evaluation of coagulation is valuable since changes in the hemostatic profile of these patients have been associated with certain complications and patients’ prognosis. Although d-dimers and fibrinogen levels are commonly elevated in patients with COVID-19, these laboratory findings cannot reliably identify patients at high risk for thromboembolism. Viscoelastic methods such as rotational thromboelastometry (ROTEM) provide a detailed insight into clot dynamics. Due to their advantageous properties, these methods have the potential to detect the level of hypercoagulability associated with increased thromboembolic risk [8]. Broad evidence exists regarding the use of viscoelastic methods to predict thromboembolic rates [13,14]. 

The purpose of this pilot study was to evaluate the hemostatic profile of patients with hip fractures and concomitant COVID-19 using rotational thromboelastometry, and to assess the impact of COVID-19 infection on the procoagulant state of patients with hip fractures. 

## 2. Material and Methods

A pilot retrospective observational study was performed from August 2020 to February 2021 at the Attikon University Hospital of Athens. COVID-19 patients with hip fractures were considered eligible for the study, while patients with congenital or acquired coagulopathies were excluded. All patients received prophylactic doses of low molecular weight heparin (LMWH). The extrinsically activated ROTEM test (EXTEM) was performed in all patients (ROTEM analyzer; Tem Innovations GmbH, Munich, Germany), as previously described [15]. The following EXTEM parameters were recorded: clotting time (CT, seconds); clot formation time (CFT, seconds); amplitude recorded at 10 min (A10, mm); a angle (a°); maximum clot firmness (MCF, mm) and lysis index at 60 min (LI60, %). The demographics and clinical parameters of included patients were reviewed, while their recorded biochemical, hematological and conventional coagulation parameters from the collected blood samples during their admission to the hospital were analyzed. The study was approved by the Institutional Review Board of Attikon University Hospital (83;01/02/2021).

To compare the hemostatic profile of patients with COVID-19 and hip fractures with the coagulopathy associated with each of these two conditions separately, we used two previously recruited groups of patients [8,11]. Specifically, 198 previously recruited non-COVID-19 patients with hip fractures represented the first comparison group showing the hemostatic profile associated with hip fractures without COVID-19 [8], while 21 previously recruited patients with mild COVID-19 (i.e., without need for admission to the intensive care unit due to COVID-19 related respiratory distress, similar severity to our newly enrolled patients) and without fractures represented the second comparison group showing the hemostatic profile of mild COVID-19 [11]. Patients in both these groups were on thromboprophylaxis with low molecular weight heparin (LMWH). 

Descriptive statistics of the study population were calculated and presented as means ± SD, medians and interquartile ranges (IQR), or as frequencies (percentages) when appropriate. Demographics, clinical characteristics, conventional laboratory values and ROTEM parameters were compared between the study groups (COVID-19 patients, non-COVID-19 hip fracture patients, and COVID-19 patients with hip fractures) using the Chi-square test for categorical variables, and the Wilcoxon rank-sum (Mann–Whitney) test or the Kruskal–Wallis test for continuous variables. Multiple linear regression analysis was also performed to evaluate the independent impact of COVID-19 on the hemostatic profile of patients with hip fractures as reflected by ROTEM parameters, adjusted for age, gender, Charlson Comorbidity Index (CCI) and Body Mass Index (BMI). Stata 15.0 software was used for analysis (Stata Corp., College Station, TX, USA). For all tests, a *p*-value < 0.05 indicates statistical significance.

## 3. Results

The final study population consisted of 20 COVID-19 patients with hip fractures, while one patient was excluded due to V Leiden disease. All patients were included in the study before surgery. All patients tested positive for COVID-19 with real-time reverse-transcriptase-polymerase chain reaction (rRT-PCR) assay on admission to the hospital, while COVID-19 was considered of mild severity in all patients, and none of them needed admission to the intensive care unit due to COVID-19 related respiratory distress. The median age of the COVID-19 patients with hip fractures was 79 years (interquartile range [IQR], 72–80). There were 11 (55%) men and 9 (45%) women. COVID-19 and non-COVID-19 hip fracture patients had similar CCI (*p* = 0.53) and BMI (*p* = 0.61). Three COVID-19 patients with hip fractures presented with thromboembolic complications: two with pulmonary embolism, and one with deep vein thrombosis. The demographic and clinical parameters of the three study groups (COVID-19 patients, non-COVID-19 hip fracture patients, and COVID-19 hip fracture patients) are summarized in Table 1.

COVID-19 hip fracture patients and non-COVID-19 hip fracture patients had similar platelet counts (medians: 200 × 10^3^/mL vs. 219 × 10^3^/mL, *p* = 0.19) and Prothrombin Time (medians: 12.8 s vs. 12.2 s, *p* = 0.86), while the activated Partial Thromboplastin Time (aPTT) was prolonged in hip fracture patients with COVID-19 (medians: 39.5 s vs. 31.4 s, *p* < 0.001). Although prolongation of aPTT is not consistent with the procoagulant effect of COVID-19, the same finding has been achieved in other studies as well, where aPTT was prolonged due to COVID-19 [16,17]. Moreover, COVID-19 was associated with elevated d-dimers and fibrinogen levels since fibrinogen (medians: 4.6 g/L vs. 3.4 g/L, *p* < 0.001) and d-dimers (medians: 2.66 ng × 10^3^/mL vs. 1.85 ng × 10^3^/mL, *p* < 0.001) were higher in hip fracture patients with COVID-19 compared to hip fracture patients without COVID-19. The conventional laboratory findings of the 3 study groups (COVID-19 patients, non-COVID-19 hip fracture patients, and COVID-19 hip fracture patients) are summarized in Table 2. Additionally, the median PLT count for the three patients with thromboembolic events was 208 × 10^3^/mL (IQR, 155–265), their median prothrombin time was 11.5 s (IQR, 10.4–12.3), their median Partial Thromboplastin Time was 37.7 s (IQR, 35.8–44.2), their median d-dimers level was 2.45 ng × 10^3^/mL (IQR, 2.18–2.74), and their median fibrinogen value was 4.4 g/L (IQR, 4.3–5.2). Due to the small number of patients with and without thromboembolic events, comparisons of these parameters are of limited value.

Regarding the EXTEM findings, COVID-19 hip fracture patients had a higher amplitude of clot firmness at 10 min (medians: 70.0 mm vs. 54.0 mm, *p* < 0.001), maximum clot firmness (medians: 75.5 mm vs 65.0 mm, *p* < 0.001), alpha angle (medians: 80.0° vs. 74.0°, *p* < 0.001) and lysis index at 60 min (medians: 99.0% vs. 91.0%, *p* < 0.001) values than non-COVID-19 hip fracture patients, indicating a procoagulant activity and impaired fibrinolysis due to COVID-19 (Table 3). Interestingly, the median value of lysis index at 60 min (99%) of COVID-19 patients with hip fractures was consistent with fibrinolysis shut down as defined by ROTEM parameters (EXTEM LI60 values ≥98%) [18,19]. Moreover, COVID-19 hip fracture patients had also higher amplitude of clot firmness at 10 min (medians: 70.0 mm vs. 66.0 mm, *p* = 0.008), maximum clot firmness (medians: 75.5 mm vs. 72.0 mm, *p* = 0.025) and lysis index at 60 min (medians: 99.0% vs. 97.0%, *p* < 0.001) compared to COVID-19 patients without hip fractures, which also indicates that the traumatic impact of hip fractures contributes to the prothrombotic state. Furthermore, COVID-19 hip fracture patients had shorter clot formation time values compared to COVID-19 patients without hip fractures (medians: 44.0 s vs. 48.0 s, *p* < 0.001) and to non-COVID-19 hip fracture patients (medians: 40.0 s vs. 84.5 s, *p* < 0.001). Finally, clotting time was similar between COVID-19 and non-COVID-19 hip fracture patients (medians: 61.0 s vs. 60.0 s, *p* = 0.57), while COVID-19 hip fracture patients had shorter clotting time compared to COVID-19 patients without hip fractures (medians: 61.0 s vs. 71.0 s, *p* < 0.001). The median clotting time for the three patients with thromboembolic events was 59 s (IQR, 58–62), their median clot formation time was 43 s (IQR, 42–48), their median amplitude of clot firmness at 10 min was 71 mm (IQR, 67–72), their median maximum clot firmness was 80 mm (IQR, 79–85), and their median lysis index at 60 min was 98% (IQR, 98–99). Although these values indicate that patients who develop thromboembolic events have a hypercoagulable profile, statistical comparison of ROTEM parameters between patients with and without thromboembolic complications is of very limited value due to the small number of patients.

Multivariable linear regression analysis (adjusted for gender, age, BMI, and CCI index) demonstrated that COVID-19 was independently associated with increased amplitude of clot firmness at 10 min (coefficient: 15.8, 95% CI: 13.6–18.1; *p* < 0.001), maximum clot firmness (coefficient: 10.0, 95% CI: 7.4–12.5; *p* < 0.001), lysis index at 60 min (coefficient: 6.8, 95% CI: 4.3–9.3; *p* < 0.001) and alpha angle (coefficient: 6.1, 95% CI: 3.9–8.4; *p* < 0.001) in hip fracture patients, while COVID-19 significantly shortened clot formation time (coefficient: −39.9, 95% CI: −43.4 to −36.6; *p* < 0.001). The results of regression analysis further confirmed the direct relationship of COVID-19 with hypercoagulability and fibrinolysis shutdown in patients with hip fractures (Table 4). 

## 4. Discussion

The impact of COVID-19 on the prognosis of patients with hip fractures has been evaluated in a few studies indicating that COVID-19 in hip fracture patients is associated with a seven- to 10-fold increased mortality risk [5,20,21,22]. The also markedly increased complication rate contributes to the low survival of these patients. The reported incidence of thromboembolic complications in COVID-19 positive patients with hip fractures under thromboprophylaxis is 6.5–13.5%, while the respective incidence after hip fractures without COVID-19 is only about 1.5–2.0% [23,24,25,26]. Our results are in line with these findings since the rate of thromboembolic events in our study was also 15% (3 of 20 patients). Based on the results of our study, the significantly higher thrombotic risk in COVID-19 patients with hip fractures can be attributed to the increased clot strength and fibrinolysis shutdown, as shown by ROTEM findings. More specifically, COVID-19 was independently associated with increased amplitude of clot firmness at 10 min, maximum clot firmness, lysis index at 60 min and alpha angle in hip fracture patients, while COVID-19 significantly shortened clot formation time. The increased thromboembolic risk due to COVID-19 highlights the importance of changes in the thromboprophylactic protocol of these patients, therefore further clinical research is warranted to ascertain the need for modification of the current thromboprophylactic protocol, including individualized anticoagulation based on ROTEM findings, increase in the anticoagulation dose or administration of fibrinolytic agents.

COVID-19 resulted in enhanced clot strength and firmness as shown by the higher A10 and MCF parameters in COVID-19 patients with hip fractures. The same effect of COVID-19 on clot dynamics has been also demonstrated in other studies using viscoelastic methods to investigate the nature of hemostatic disturbances due to COVID-19 associated coagulopathy [27,28]. In a recent systematic review reporting the outcomes of five studies with TEG results and five studies with ROTEM results in COVID-19 patients, increased clot strength was apparent in all TEG studies as shown by the increased maximum amplitude, a thromboelastographic parameter that reflects enhanced clot strength. In 4 out of the 5 ROTEM studies, high MCF values were also reported. A possible cause for the increased clot strength due to COVID-19 is increased thrombin generation due to endothelial damage, as shown by the elevated endogenous thrombin potential (ETP) in COVID-19 patients [28,29]. Increased clot strength resulting in a hypercoagulable state has been also demonstrated in patients with hip fractures [14]. The combining effect of COVID-19 associated coagulopathy and hip fracture coagulopathy on clot firmness may be one of the major causes for increased thromboembolic incidence in this population [8,30,31]. Although CFT was shortened in our COVID-19 patients with hip fractures, indicating an accelerated clot formation dynamic due to COVID-19, CT was similar between COVID-19 and non-COVID-19 patients. A similar finding was highlighted by another systematic review that investigated the role of TEG analysis in COVID-19 patients, with most of the included studies reporting a normal R-time, a TEG parameter similar to CT that corresponds to the induction of coagulation, showing that this parameter is less consistent to reveal hypercoagulability in this clinical setting [32].

Another important finding regarding the effect of COVID-19 on the hemostatic mechanisms of patients with hip fractures was fibrinolysis shutdown. Impaired fibrinolysis has been also identified as a significant cause for increased thromboembolic risk in COVID-19 patients in other studies [9,10]. Elevated plasminogen activator inhibitor-1 (PAI-1) activity has been hypothesized as one of the major causes for COVID-19 associated fibrinolysis resistance and fibrinolysis shutdown in COVID-19 patients [29]. In a single-center cohort study of 40 critically ill patients with COVID-19, Kruse et al. also found signs of severe hypofibrinolysis using ROTEM analysis. The authors of this study reported that maximum lysis (ML), a ROTEM parameter for the evaluation of fibrinolysis similar to LI60, was lower in patients with thromboembolic events compared to patients without thromboembolic events [9]. Moreover, they showed that combining values of ROTEM parameters for hypofibrinolysis with elevated D-dimer levels had a high prediction accuracy for thromboembolic complications. A similar finding was also shown by Wright et al., who studied the hemostatic profile of 44 critically-ill COVID-19 patients using TEG and found a complete fibrinolysis shutdown in 57% of their patients, while low LY30, a parameter similar to LI60 was associated with thromboembolic events [10]. The incidence of complete shutdown in our study cohort was 80% (16 of 20 patients), higher than that reported by Wright et al. which can be attributed to the combining effect of hip fractures and COVID-19.

There are some limitations of our study that must be acknowledged. First, the relatively small number of patients does not allow us to draw any firm conclusions regarding the clinical implications of the identified hypercoagulability due to COVID-19, since comparisons of patients with and without VTE events would provide results of very limited strength. However, although this is a pilot study with a small number of patients, our findings are in line with similar findings of other studies regarding the prothrombotic state of COVID-19. Additionally, due to the use of two previously recruited groups, the three analyzed groups in this study were not matched for certain demographic and clinical parameters. However, logistic regression was performed to address the issue of potential confounding factors of the relationship between COVID-19 and ROTEM findings in hip fracture patients. Information about the mortality or complication rate of the studied population is also limited by the short follow up of the study, since it is possible that a larger number of patients than the one reported presented with thromboembolic complications in the following weeks. Also, hospital records for some clinical parameters (e.g., comorbidities, medications) and laboratory assays (inflammatory markers, INTEM and APTEM assays) for the included patients were limited, which did not allow any comparison of these parameters among the three studied groups. Last, other laboratory studies of coagulation to confirm and further investigate the identified mechanisms responsible for the hypercoagulability seen in our study group were not performed. Specifically, plasminogen activator inhibitor-1 (PAI-1) activity and endogenous thrombin potential (ETP) were not determined, two laboratory variables that could further elucidate the pathophysiology of the observed fibrinolysis shutdown and enhanced clot firmness, respectively.

## 5. Conclusions 

Conclusively, COVID-19 has a detrimental impact on the already increased thrombotic risk of patients with hip fractures, as shown by the high reported rate of thromboembolic complications in this population, which was also evident in our study. Our study shed light on the pathophysiology involved in the hypercoagulability associated with the increased thrombotic risk of these patients. The main causes for the coagulopathy associated with a higher thrombotic risk in COVID-19 patients with hip fractures include increased clot strength and fibrinolysis shutdown as shown by the altered ROTEM findings. These hemostatic alterations cannot be detected by conventional coagulation studies, while elevated fibrinogen levels cannot reliably detect the hemostatic profile associated with thromboembolic events. 

## Figures and Tables

**Table 1:** Characteristics of the study population.

	Hip Fracture Patients (Group A = 198)	Patients with Mild COVID-19 (Group B = 21)	Hip Fracture Patients with COVID-19 (Group C = 20)	Overall *p*	A vs. B	A vs. C	B vs. C
Age (years)	78.9 ± 5.2, 80 (76–82)	68.2 ± 20.4, 73.0 (50.0–88.0)	77.2 ± 4.9, 79.0 (72.0–80.0)	0.16	0.13	0.20	0.45
Gender (males %)	93 (46.9)	11 (52.3)	11 (55)	0.72	0.22	0.46	0.86
CCI	5.9 ± 1.2, 6.0 (5.0–7.0)	-	6.1 ± 0.9, 6.0 (5.5–7.0)	0.53	N/A	0.53	N/A
BMI (Kg/m^2^)	24.6 ± 3.6, 24 (22–27)	-	24.0 ± 2.5, 24.5 (22–26)	0.61	N/A	0.61	N/A
Creatinine (mg/dL)	1.18 ± 0.36,1.09 (1.01–1.19)	0.97 ± 0.52, 0.90 (0.70–1.00)	1.29 ± 0.21, 1.20 (1.15–1.35)	<0.001	<0.001	<0.001	<0.001

Data are presented as mean ± SD; median and interquartile range. The Kruskal-Wallis test was used for the overall comparison, while the Mann-Whitney test was used for the pairwise comparisons. Abbreviations: BMI, Body Mass Index; CCI, Charlson Comorbidity Index.

**Table 2:** Conventional coagulation assays of the study cohort.

Variables	Hip Fracture Patients (Group A = 198)	Patients with Mild COVID-19 (Group B = 21)	Hip Fracture Patients with COVID-19 (Group C = 20)	Overall *p*	A vs. B	A vs. C	B vs. C
PLTs (count × 10^3^/mL)	218.9 ± 48.0, 216.0 (180.0–245.0)	285.6 ± 120.2, 253.0 (207.0–396.0)	200.0 ± 32.7, 201.0 (189.0–211.0)	<0.001	0.015	0.19	0.014
aPTT (s)	31.4 ± 3.9, 31.0 (29.0–34.0)	39.2 ± 6.7, 37.8 (34.3–41.9)	39.5 ± 3.3, 39.5 (37.0–42.4)	<0.001	<0.001	<0.001	0.35
PT (s)	12.2 ± 2.0, 12.0 (11.0–14.0)	13.5 ± 1.9, 13.6 (12.3–14.2)	12.8 ± 2.9, 12.1 (10.8–14.7)	0.10	0.030	0.86	0.24
Fibrinogen (g/L)	3.4 ± 0.5, 3.5 (3.2–3.7)	4.4 ± 1.3, 4.3 (3.9–4.9)	4.6 ± 0.3, 4.6 (4.3–4.9)	<0.001	0.001	0.001	0.17
d-dimers (ng × 10^3^/mL)	1.85 ± 0.53, 1.85 (1.50–2.17)	1.32 ± 1.28, 0.86 (0.54–1.21)	2.65 ± 0.65, 2.66 (2.31–3.03)	<0.001	<0.001	<0.001	<0.001

Data are presented as mean ± SD; median and interquartile range. The Kruskal–Wallis test was used for the overall comparison, while the Mann-Whitney test was used for the pairwise comparisons. Abbreviations: PLTs, platelets; PTT, activated partial thromboplastin time; PT, prothrombin time; INR, international normalized ratio.

**Table 3:** Extrinsically activated rotational thromboelastometry test (EΧΤΕΜ) parameters among the study groups.

Variables	Hip Fracture Patients (Group A = 198)	Patients with Mild COVID-19 (Group B = 21)	Hip Fracture Patients with COVID-19 (Group C = 20)	Overall *p*	A vs. B	A vs. C	B vs. C
EXTEM CT (s)	59.8 ± 6.6, 60.0 (56.0–64.0)	73.5 ± 12.0, 71.0 (66.0–81.0)	61.0 ± 5.3, 61.0 (58.0–63.5)	<0.001	<0.001	0.57	<0.001
EXTEM CFT (s)	84.0 ± 7.4, 84.5 (81.0–88.0)	59.5 ± 24.9, 48.0 (45.0–61.0)	44.4 ± 4.2, 44.0 (41.0–47.0)	<0.001	<0.001	<0.001	0.019
EXTEM A10 (mm)	53.4 ± 4.6, 54.0 (51.0–57.0)	65.0 ± 5.4, 66.0 (63.0–69.0)	69.3 ± 5.9, 70.0 (67.0–72.5)	<0.001	<0.001	<0.001	0.008
EXTEM MCF (mm)	65.8 ± 5.6, 65.0 (63.0–69.0)	72.4 ± 4.0, 72.0 (70.0–74.0)	75.9 ± 5.1, 75.5 (72.5–79.5)	<0.0001	<0.001	<0.001	0.025
EXTEM Alpha angle (°)	73.7 ± 4.8, 74.0 (71.0–77.0)	78.1 ± 4.7, 80.0 (78.0–81.0.)	80.0 ± 5.2, 80.0 (76.0–84.0.)	<0.001	<0.001	<0.001	0.43
EXTEM LI60 (%)	91.6 ± 5.4, 91.0 (88.0–95.0)	96.3 ± 2.9, 97.0 (94.0–99.0)	98.5 ± 1.2, 99.0 (98.0–99.5)	<0.001	<0.001	<0.001	0.011

Data are presented as mean ± SD; median and interquartile range. The Kruskal-Wallis test was used for the overall comparison, while the Mann-Whitney test was used for the pairwise comparisons. Abbreviations: CT, clotting time; CFT, clot formation time; A10, clot amplitude at 10 min; MCF, maximum clot firmness; LI60, lysis index at 60 min.

**Table 4:** Results of multivariable regression analysis for the evaluation of the effect of COVID on the coagulation profile of patients with hip fractures as evaluated by ROTEM parameters, adjusted for age, gender, Charlson Comorbidity Index and Body Mass Index. In the model, presence of COVID-19 disease is used as an independent variable and ROTEM parameters as dependent variables.

ROTEM Parameters	Presence of COVID-19 in Patients with Hip Fracture		
	Coefficient	95% CI	*p*-Value
EXTEM CFT (s)	−39.9	−43.3 to −36.6	<0.001
EXTEM MCF (mm)	10.0	7.4–12.5	<0.001
EXTEM A10 (mm)	15.8	13.6–18.1	<0.001
EXTEM LI60 (%)	6.8	4.3–9.3	<0.001
EXTEM alpha angle (°)	6.1	3.9–8.4	<0.001

Abbreviations: CI, confidence interval, CT, clotting time; CFT, clot formation time; A10, clot amplitude at 10 min; MCF, maximum clot firmness; LI60, lysis index at 60 min.

## Data Availability

The data presented in this study are available upon reasonable request to the corresponding authors.
